# Survey of echocardiography provision and practice in ICUs in the United Kingdom

**DOI:** 10.1186/cc9446

**Published:** 2011-03-11

**Authors:** A Cooke, S Bruemmer-Smith, J McLoughlin, J McCaffrey

**Affiliations:** 1 Belfast City Hospital, Belfast, UK; 2 Brighton and Sussex University Hospital, Brighton, UK; 3 Sir Charles Gairdner Hospital, Perth, Australia

## Introduction

Echocardiography in the intensive care unit (ITU) has been shown to be a valuable aid to clinical decision-making [1-3]. Currently, there is no formal training process for intensivists wishing to learn echocardiography in the United Kingdom, and there is little information on the current state of clinical practice.

## Methods

A structured questionnaire was sent to each intensive care unit in the United Kingdom. The questionnaire detailed information regarding the availability of echocardiography and the frequency that echocardiograms are performed in the ITU. We enquired after the level of training in echocardiography by intensivists, the reporting process and availability of currently provided training. Opinions on the necessity of formalised training and the level of that training were also sought.

## Results

Responses were obtained from 32 units ranging in size from five to 35 critical care beds. A total of 53.13% have their own dedicated echo machine. Only 15.6% have a transoesophageal probe. In 28% of ITUs echocardiograms are performed by intensivists; however, only 25% of ITUs currently offer echocardiography training to intensive care trainees. Seventy-eight per cent of respondents believed that ITU physicians should have at least intermediate echocardiography skills; 97% respondents believed that a national training programme should be established for echocardiography practice by ITU physicians.

## Conclusions

Echocardiography is currently widely used in ITUs throughout the United Kingdom but is often being performed by physicians with little or no formal training. There is almost unanimous support for a national structure and a formalised curriculum to achieve safe widespread training.

## References

[B1] Orme RM Br J Anaesth 2009 102 340 344 10.1093/bja/aen378 19151420

[B2] Breitkreutz R Minerva Anesth 2009 75 285 292 19412146

[B3] Price S Intensive Care Med 2006 32 48 59 10.1007/s00134-005-2834-7 16292626

